# Essential Role of IFN-γ in Regulating Gut Antimicrobial Peptides and Microbiota to Protect Against Alcohol-Induced Bacterial Translocation and Hepatic Inflammation in Mice

**DOI:** 10.3389/fphys.2020.629141

**Published:** 2021-01-18

**Authors:** Ruichao Yue, Xiaoyuan Wei, Jiangchao Zhao, Zhanxiang Zhou, Wei Zhong

**Affiliations:** ^1^Center for Translational Biomedical Research, University of North Carolina at Greensboro, North Carolina Research Campus, Kannapolis, NC, United States; ^2^Division of Agriculture, Department of Animal Science, University of Arkansas, Fayetteville, AR, United States; ^3^Department of Nutrition, University of North Carolina at Greensboro, North Carolina Research Campus, Kannapolis, NC, United States

**Keywords:** alcohol, IFN-γ, STAT, antimicrobial peptide, microbiota, PAMP translocation

## Abstract

The mechanisms by which alcohol provokes bacterial translocation in the development of alcoholic liver disease (ALD) remain incompletely defined. Our previous study demonstrates that impaired gut epithelial antimicrobial defense is critically involved in the pathogenesis of ALD. The study was set to determine the mechanisms of how alcohol inhibits the antimicrobial ability of intestinal epithelial cells (IECs) and to explore possible solutions to this issue. C57BL/6J mice were fed either alcohol or isocaloric dextrin liquid diet for 8 weeks, and intestinal IFN-γ-signal transducer and activator of transcription (STAT) signaling was analyzed. We found that chronic alcohol exposure led to a significant reduction in intestinal IFN-γ levels compared to a control; the protein levels of phosphorylated STAT1 (p-STAT1) and p-STAT3 were both declined by alcohol. We then tested the effects of IFN-γ-STAT signaling on regulating antimicrobial peptides (AMPs), gut microbiota, and disease progression of ALD in a mouse model of chronic alcohol feeding, time-course acute IFN-γ treatment, and *in vivo* and *in vitro* IEC-specific STAT1 or STAT3 knockout mouse models, respectively. Administration of IFN-γ activated intestinal STAT1 and STAT3, upregulated the expression of Reg3 and α-defensins, orchestrated gut microbiota, and reversed alcohol-induced intestinal ZO-1 disruption and systemic endotoxin elevation as well as hepatic inflammation. Meanwhile, acute IFN-γ treatment time-dependently induced AMP expression and α-defensin activation. We then dissected the roles of STAT1 and STAT3 in this progress. Lack of IEC-specific STAT3 inhibited IFN-γ-induced expression of Reg3 and α-defensins and hindered activation of α-defensins *via* inactivating matrix metallopeptidase 7 (MMP7), whereas lack of IEC-specific STAT1 impaired IFN-γ-stimulated expression of α-defensins and the IEC marker, sodium-hydrogen exchanger 3. Lastly, we found that interleukin (IL)-18, a known IFN-γ inducer, was also reduced by alcohol in mice. IL-18 treatment to alcohol-fed mice normalized gut IFN-γ levels and ameliorated organ damages in both the intestine and liver. Taken together, the study reveals that IFN-γ is critically involved in the regulation of AMPs through regulation of STAT1 and STAT3; impaired IFN-γ-STAT signaling provides an explanation for alcohol-induced gut antimicrobial dysfunction and microbial dysbiosis. Therefore, IFN-γ remains a promising host defense-enhancing cytokine with unexplored clinical potential in ALD therapy.

## Introduction

Alcoholicliver disease (ALD) is one of the leading causes of chronic liver disease, which encompasses a spectrum of liver pathologies ranging from steatosis, hepatitis, fibrosis, cirrhosis, and hepatocellular carcinoma (Gao and Bataller, 2011; Bajaj, 2019). Among the various factors affecting the development of ALD, the role of a disrupted gut-liver axis is very crucial. In general, alcohol consumption leads to disrupted gut barrier as well as intestinal bacterial overgrowth and enteric dysbiosis. These factors contribute to the increased translocation of microbial products, namely pathogen associated molecular patterns (PAMPs), to the liver and subsequent alcohol-related damage (Yan et al., 2011; Bull-Otterson et al., 2013; Zhong et al., 2019). One well-known PAMP is lipopolysaccharide (LPS), levels of which have been shown to correlate with the severity of liver damage in patients with alcoholic hepatitis (Hanck et al., 1998; Parlesak et al., 2000). Mice deficient in LPS receptor Toll-like receptor 4 (TLR4), CD14, or downstream signaling molecule, MyD88, are resistant to ALD (Uesugi et al., 2001; Yin et al., 2001; Hritz et al., 2008).

There has been growing emphasis on the importance of functional antimicrobial peptides (AMPs) in protecting the gut and liver from alcohol-induced enteric dysbiosis and damage. AMPs are produced and secreted by intestinal epithelial cells and Paneth cells and serve as important innate immune regulators in maintaining gut microbial growth and composition (Bevins and Salzman, 2011; Muniz et al., 2012). Chronic alcohol feeding reduces intestinal AMPs, including C-type lectins (Reg3β and Reg3γ; Yan et al., 2011; Wang et al., 2016), α-defensins (Zhong et al., 2019), and cathelicidin-related antimicrobial peptide (CRAMP; He et al., 2019). Moreover, overexpression of Reg3γ in mice restricted bacterial colonization and ameliorated alcohol-induced steatohepatitis (Wang et al., 2016). We previously reported alcohol-induced Paneth cell antimicrobial dysfunction and protection from ALD by human α-defensin 5 administration in mice (Zhong et al., 2019). However, it remains unclear how alcohol intoxication impairs the host antimicrobial defense system and whether such mechanism could be manipulated to combat abnormal gut microflora and ALD progression.

IFN-γ is a type II IFN cytokine critical to both innate and adaptive immunity, and helps fight against viral and bacterial infections (Ma et al., 2011). Most of the studies that established the role of IFN-γ in bacterial infection are based on the regulation of the host’s ability to respond to bacteria. Treatment of animal cells with IFN-γ leads to changes in target gene expression, including an AMP – cathelicidin (Shtrichman and Samuel, 2001; Fabri et al., 2011). IFN-γ also acts as a direct intestinal secretagogue for both Paneth and goblet cells to release AMP and mucus (Farin et al., 2014). Furthermore, IFN-γ plays an essential role in intestinal epithelial cell proliferation and apoptosis through regulating β-catenin pathway (Nava et al., 2010). The downstream signaling of IFN-γ involves signal transducer and activator of transcription (STAT) proteins, including STAT1, STAT3, and STAT5 (van Boxel-Dezaire and Stark, 2007; Ruiz et al., 2015). Intestinal STATs have an essential role in the host antimicrobial responses (Gavrilescu et al., 2004; Lieberman et al., 2004; Ching et al., 2018). Mice deficient in STAT1 are highly susceptible to infections caused by bacterial pathogens and viruses (Meraz et al., 1996). STAT3 transcriptionally regulates the expression of a variety of molecules with antimicrobial ability, including AMPs (Choi et al., 2013; Wittkopf et al., 2015).

Taken together, these studies indicate an important role for IFN-γ-STAT signaling in intestinal antimicrobial defense and integrity, which necessitates investigation in the context of ALD. Here, we investigated whether aberrant IFN-γ-STAT signaling would be a causal factor driving alcohol-induced AMP dysfunction, gut microbiome dysbiosis, PAMP translocation, and hepatic inflammation. Mice deficient in intestinal epithelial cell (IEC)-specific STAT1 or STAT3 were used to dissect the role of STATs in regulating AMPs. We also tested the therapeutic effects of an IFN-γ inducer, IL-18, to protect against alcohol-induced intestinal and hepatic damage in mice.

## Materials and Methods

### Mice

C57BL/6J wild type (WT) mice, villin-Cre transgenic mice (stock no. 004586), Stat1 floxed mice (Stat1^flox/flox^; stock no. 012901), and Stat3 floxed mice (Stat3^flox/flox^; stock no. 016903), were purchased from the Jackson Laboratory (Bar Harbor, ME, United States). Conditional knockout mice with Stat1 or Stat3 deletion in IECs (Stat1^IEC−/−^ or Stat3^IEC−/−^) were created using villin promoter driving Cre recombinase in mouse with floxed P sites in the introns of Stat1 or Stat3 gene (Stat1^flox/flox^ or Stat3^flox/flox^ mouse). Mice were handled and all experiments were performed in accordance with the protocol approved by the North Carolina Research Campus Institutional Animal Care and Use Committee (project no. 19017).

### Chronic Alcohol Feeding and Treatments

For chronic alcohol feeding experiments, male C57BL/6J WT mice at 12-week-old were administrated with ethanol-containing Lieber-DeCarli liquid diet [alcohol-fed (AF)] or an isocaloric control liquid diet [pair-fed (PF)] for 8 weeks as previously described (Zhong et al., 2015). Recombinant mouse IFN-γ (Biolegend, San Diego, CA, United States; 575,308) or mouse IL-18 (MBL International Corporation, Woburn, MA, United States; B004-5) was given to AF mice at 100 ng/mouse (IFN-γ) and 1 μg/mouse (IL-18), respectively, every other day through intraperitoneal injection for the last 2 weeks.

For the acute time course experiment, male WT mice were intraperitoneally treated with a single dose of either recombinant mouse IFN-γ at 100 ng/mouse or same volume of saline. The mice were sacrificed 1, 3, or 8 h after injection.

### Mouse Small Intestinal Organoid Culture and Treatments

Small intestinal organoids were established from isolated crypts of the proximal small intestine of Stat1^flox/flox^, Stat1^IEC−/−^, Stat3^flox/flox^, or Stat3^IEC−/−^ mice as described (Sato et al., 2009) and grown with crypt niche factors and Matrigel (Corning, Corning, NY, United States). Organoids were cultured in mouse IntestiCult organoid growth medium (Stemcell Technologies, Cambridge, MA, United States) and passaged at a 1:5 ratio. Morphology of organoids, including budding and total area of the organoid cultures, was examined by light microscope during culture.

Organoid cells at 6 days after passage were treated with 1 ng/ml IFN-γ for 3 h and harvested for quantitative PCR (qPCR) using RNeasy Mini Kit (Qiagen) or immunofluorescence (IF) staining by fixing with 2% paraformaldehyde with 0.1% glutaraldehyde.

### Cecal Microbial Community Analysis

Cecal content samples were collected after feeding experiment and were stored at −80°C until further processing. Microbial DNA was extracted with DNeasy PowerLyzer PowerSoil kit (Qiagen, Germantown, MD, United States) according to the manufacturer’s instructions. The V4 region of the bacterial 16S rRNA gene was amplified and sequenced on Illumina MiSeq platform using the MiSeq Reagent kit v2 (Illumina, Inc., SanDiego, CA, United States). Mothur software package (v.1.39.5) was used to analyze the 16S rRNA MiSeq data (Schloss et al., 2009). After quality-filtering and alignment against SILVA v132 database (Quast et al., 2013), sequences were clustered into operational taxonomic units (OTU) with 97% similarity and were classified against the Ribosomal Database Project (Cole et al., 2009). The number of observed OTUs was calculated to measure alpha diversity. Bray-Curtis distance metrics were calculated to explore the dissimilarities in community structure. The analysis of similarity (ANOSIM) test was used to determine whether there is a significant difference between groups in beta diversity. Linear discriminant analysis effect size (LEfSe)^1^ was performed on the complete sequence data (no OTU threshold) to detect differentially abundant taxa among groups (Segata et al., 2011). Phylogenetic investigation of communities by reconstruction of unobserved states (PICRUSt) was used to predict the functional gene content in the cecal microbiota based on taxonomy obtained from the Greengenes reference database 13.5 (Langille et al., 2013). LEfSe analysis was then applied to explore functional gene with significantly different abundances between groups.

### Quantitative Reverse-Transcriptase PCR

Total RNA was extracted from ileal tissue using TRIzol™ reagent (Thermo Fisher Scientific) according to the manufacturer’s direction. Complimentary DNA was generated using TaqMan Reverse Transcription Reagents (Thermo Fisher Scientific). Real-time PCR was performed with SYBR green PCR master mix (Qiagen, Germantown, MD, United States) using the QuantStudio 5 real time reverse-transcriptase PCR (RT-PCR) system. Primers were designed and synthesized by Integrated DNA Technologies (Coralville, CA, United States). Primers used for qPCR were listed in Table 1. All data were normalized to the expression of 18S rRNA gene and calculated using the 2^-ΔΔCt^ method (Livak and Schmittgen, 2001) setting the values of PF as 1.

**Table 1:** Primer sequences used for quantitative PCR (qPCR) analysis.

Gene	Genebank accession number	Forward primer (5'-3')/reverse primer (5'-3')	Amplicon size
Ifng	NM_008337	CTCTTCCTCATGGCTGTTTCT TTCTTCCACATCTATGCCACTT	105 bp
Defa2	NM_001195634	AGACACTTGTCCTCCTCTCT CTGCCTGCTCCTCAGTATTAG	102 bp
Defa4	NM_010039	CCAGGGGAAGATGACCAGGCTG TGCAGCGACGATTTCTACAAAGGC	110 bp
Defa5	NM_007851	CAGGCTGATCCTATCCACAAA CTTGGCCTCCAAAGGAGATAG	97 bp
Defa20	NM_183268	AGACACTTGTCCTCCTCTCT GCTGCTCCTCAGTATTAGTCTC	99 bp
Reg3b	NM_011036	AATGGAGGTGGATGGGAATG CCACAGAAAGCACGGTCTAA	95 bp
Reg3g	NM_011260	TTCTCAGGTGCAAGGTGAAG GGCATAGCAATAGGAGCCATAG	97 bp
Lbp	NM_008489	CAGATCCGCAAGGACTTCTTAT CCACTGAGACCCATCTTTCTTC	85 bp
Cd14	NM_009841	CTGGCACAGAATGCCCTAAT TTCCTCCTAACAGCCCTACTC	110 bp
Ass1	NM_007494	GAAGAGCTGGTGAGCATGAA AGCCTGAGCGAGTTGATATTG	83 bp
Cxcl1	NM_030845	ACCCAAACCGAAGTCATAGCCAC ACTAGTGTTGTCAGAAGCCAGCGT	181 bp
Mcp1	NM_031530	TGCTGTCTCAGCCAGATGCAGTTA TACAGCTTCTTTGGGACACCTGCT	131 bp
Nhe3	NM_001081060	CTGGCTTCGTCTTTGTCATTTC GTTGGCCTTCACGTACTTCT	119 bp
Rn18s	NR_046237	ACGGACCAGAGCGAAAGCAT TGTCAATCCTGTCCGTGTCC	152 bp

### Plasma ALT and AST Levels

The alanine aminotransferase (ALT) and aspartate aminotransferase (AST) levels were measured with Thermo Scientific ALT/GPT reagent and AST/GOP reagent, respectively.

### Endotoxin Levels

Endotoxin levels in mouse blood and livers were tested using an ELISA-based method (EndoLISA Endotoxin Detection Kit; Biovendor, Asheville, NC, United States) as per the manufacturer’s instructions. The concentrations of endotoxin were expressed in endotoxin units (EU) per milliliter for plasma and EU per milligram liver tissue.

### Hepatic Lipids

Quantification assay of lipids was conducted by measuring the concentrations of triglyceride (TG) and free fatty acid (FFA) in liver tissues using BioVision (Milpitas, CA, United States) assay kits.

### ELISA

The levels of IFN-γ, IL-18, and IL-22 in the ileum were determined by ELASA kit purchased from R&D Systems (Minneapolis, MN, United States) following the manufacturer’s instructions.

### Acid Urea Polyacrylamide Gel Electrophoresis (AU-PAGE)

Ileal peptides were isolated using a modified procedure described previously (Wilson et al., 2015). Briefly, 5 cm fresh ileum were excised from the mouse and homogenized in 30% (v/v) acetic acid, incubated at 4°C with constant rocking overnight, and then ultracentrifuge at 100,000 × *g* at 4°C for 90 min. Resulting supernatants were lyophilized, and solubilized in loading solution (3 M urea in 5% acetic acid). Fifteen percent AU-PAGE gel was pre-electrophoresis for 90 min at 150 V. Samples were loaded into the gel for an additional 85 min at 150 V until methyl green had run off the bottom of the gel. Human α-defensin-5 (HD5; Peptide International, Louisville, KY, United States) was used as a positive control. After electrophoresis, gels were washed with double distilled water, stained with blue safe protein stain (Thermo Fisher Scientific, Rockford, IL, United States), and de-stained by double distilled water for 2 h.

### Immunohistochemistry

For the detection of phosphorated-STAT1 (Tyr701; Abcam, Cambridge, MA, United States; ab29045), phosphorated-STAT3 (Tyr705; 9145S) in the ileum and neutrophil infiltration (myeloperoxidase/MPO; Lifespan Biosciences, Seattle, WA, United States; LS-B6699) in the liver, immunohistochemistry was performed using the paraffin-embedded sections as described in our previous study (Zhong et al., 2013).

### Immunofluorescence

Immunofluorescence was applied to determine the levels of ileal IFN-γ, tight junction protein, ZO-1, and IEC marker, sodium-hydrogen exchanger 3 (NHE3). Cryostat sections of mouse ileum were incubated with anti-IFN-γ (Thermo Scientific; MM700), anti-ZO-1 (Millipore, Burlington, MA, Unites States; MABT11), or anti-NHE3 (Novus Biologicals, Centennial, CO, United States; NBP1-82574) followed by Alexa Fluor 594-conjugated donkey anti-rat IgG (Jackson ImmunoResearch Laboratories, West Grove, PA, United States). The nuclei were counterstained by 4',6-diamidino-2-phenylindole (DAPI; Thermo Fisher Scientific).

### Immunoblotting

Whole tissue protein was extracted using T-PER Tissue Protein Extraction Reagent (Fisher Scientific) containing a cocktail of protease inhibitors. Protein samples were separated by 10% SDS-PAGE, transblotted onto polyvinylidene difluoride membranes (Bio-Rad, Hercules, CA, United States), and then blocked with the following primary antibodies overnight, including anti-phosphorated-STAT1, anti-phosphorated-STAT3, anti-STAT1 (Cell Signaling Technology, Danvers, MA, United States; 9172S), anti-STAT3 (Cell Signaling Technology; 9139S), anti-matrix metallopeptidase 7 (MMP7; Cell Signaling Technology; 3801S), and anti-β-actin (Sigma-Aldrich; A5316). HRP-conjugated goat anti-rabbit IgG or goat anti-mouse IgG (Thermo Scientific) were used to amplify the signal. The immunoreactive bands were visualized by enhanced chemiluminescence (Thermo Scientific) and quantified by densitometry analysis.

### Statistics

All data are expressed as mean ± SD. The data were analyzed using GraphPad Prism software (La Jolla, CA, United States). Statistical significance was carried out using unpaired two-tailed Student’s *t*-test or one-way ANOVA followed by *post hoc* Newman-Keuls test where appropriate and considered significant at *p* < 0.05.

## Results

### Chronic Alcohol Feeding Impairs Intestinal IFN-γ-STAT Signaling

**Figure 1:**
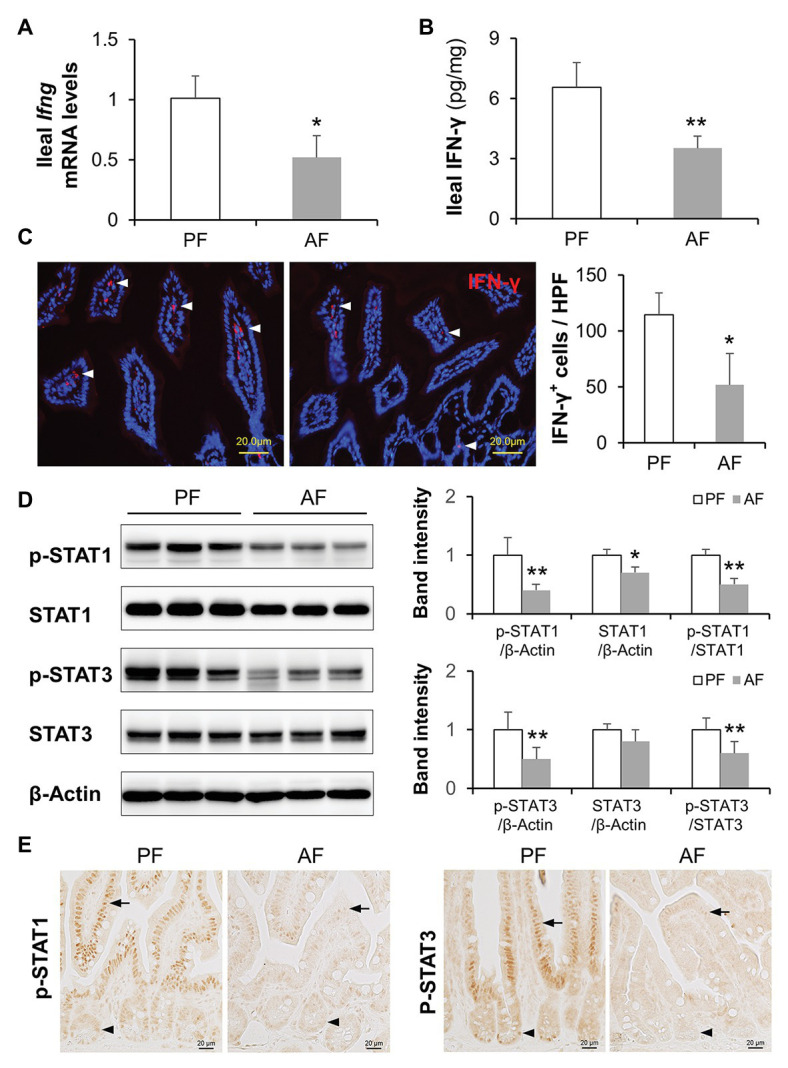
Intestinal IFN-γ-signal transducer and activator of transcription (STAT) signaling is impaired in mice fed alcohol. **(A)** mRNA levels of ileal IFN-γ (*n* = 6 per group). **(B)** Protein levels of ileal IFN-γ measured by ELISA. **(C)** Representative immunofluorescence (IF) staining of ileal IFN-γ (red). Nuclei were counterstained by 4',6-diamidino-2-phenylindole (DAPI; blue). Scale bar, 20 μm. IFN-γ positive stained cells were quantified and compared to high power field (HPF). **(D)** Western blot (WB) and quantification of ileal STAT1 and STAT3. **(E)** Representative immunohistochemistry (IHC) staining of phosphorylated STAT1 and STAT3 in the ileum of mice. ^*^p < 0.05 and ^**^p < 0.01. PF, pair-fed; AF, alcohol-fed.

Chronic alcohol feeding has been shown to reduce intestinal AMP levels, such as Reg3β and Reg3γ (Yan et al., 2011; Wang et al., 2016), α-defensins (Zhong et al., 2019), and CRAMP (He et al., 2019), in mice. We explored if IFN-γ is involved in alcohol-induced AMP reduction using a mouse model of ALD. Intestinal IFN-γ levels were significantly lower in AF group than in PF group as indicated at both mRNA and protein levels (Figures 1A,B). This observation was further confirmed by IF staining of IFN-γ; fair amount of positive staining was detected in intestinal lamina propria of PF mice where IFN-γ-producing immune cells reside, whereas fewer positive cells were apparent in AF group (Figure 1C). In accordance with lower IFN-γ levels, AF mice had a 50–60% reduction in ileal phosphorylated STAT1 and STAT3 compared with PF mice. Total STAT1 but not total STAT3 was also slightly decreased by alcohol (Figure 1D). Immunohistochemical staining showed that alcohol reduced positive staining of phosphorylated STAT1 (p-STAT1) and p-STAT3 in both IECs (Figure 1E; arrows) and crypt cells (Figure 1E; arrowheads). These findings indicate a defective IFN-γ-STAT signaling in the intestine after chronic alcohol exposure.

### Restitution of IFN-γ Ameliorates Alcohol-Induced Hepatic Inflammation in Mice

**Figure 2:**
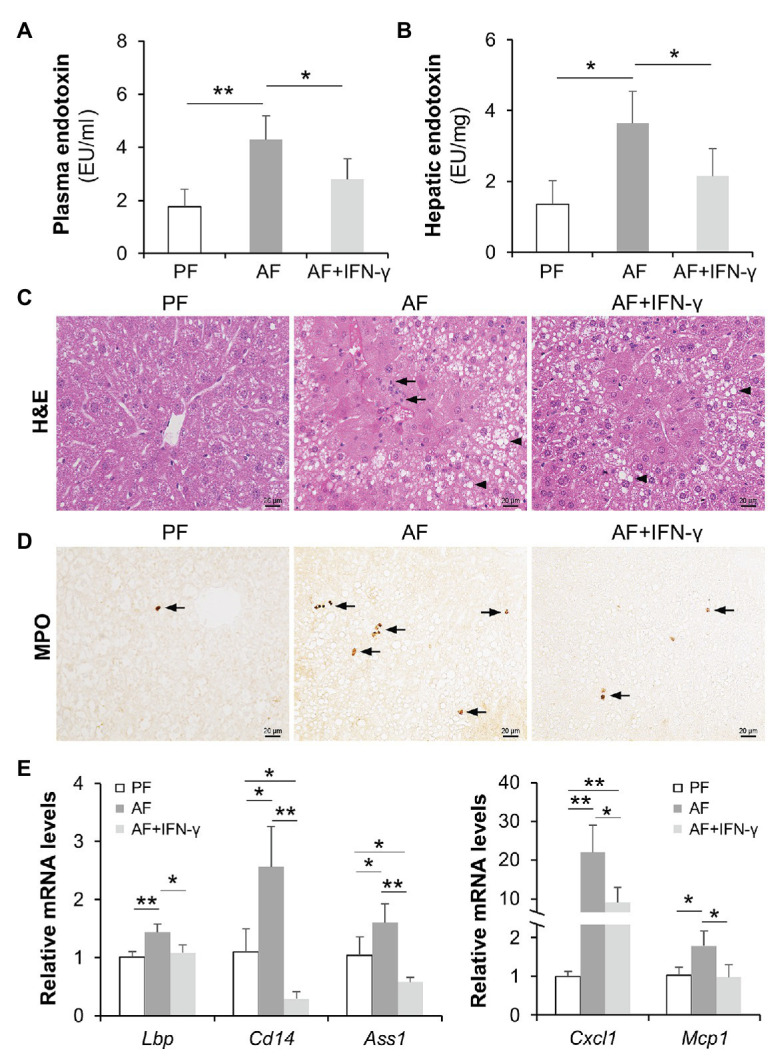
IFN-γ treatment reduces alcohol-induced lipopolysaccharide (LPS) translocation and hepatic inflammation. **(A)** Plasma endotoxin levels (*n* = 8 per group). **(B)** Hepatic endotoxin levels. **(C)** Hematoxylin and eosin (H&E) staining of mouse liver sections. Arrowheads indicate lipid accumulation and arrows indicate inflammatory cells. Scale bar, 20 μm. **(D)** Representative IHC staining of neutrophil marker, MPO, in the liver of mice. Arrows indicate positive staining. Scale bar, 20 μm. **(E)** mRNA levels of hepatic LPS signaling molecules and inflammatory chemokines. ^*^p < 0.05 and ^**^p < 0.01. PF, pair-fed; AF, alcohol-fed.

To determine if the reduction of intestinal IFN-γ levels is linked to alcohol-induced hepatic inflammation possibly through PAMP translocation, we administrated AF mice with recombinant IFN-γ to restore IFN-γ levels and evaluated systemic LPS levels as well as hepatic inflammatory responses. As shown in Figures 2A,B, alcohol-elevated plasma and hepatic LPS levels were both blunted by IFN-γ treatment. Accumulation of infiltrated inflammatory cells was more frequently observed in AF group compared to that in PF or AF+IFN-γ group (Figure 2C; arrows). IFN-γ treatment did not alter the amount of lipids accumulated in the liver (Figure 2C; arrowheads). Staining of myeloperoxidase (MPO), a heme-containing peroxidase expressed mainly by neutrophils, showed that IFN-γ treatment resulted in a compelling reduction of alcohol-induced hepatic neutrophil infiltration (Figure 2D). We further analyzed expressions of hepatic LPS signaling molecules and cytokines. Alcohol exposure significantly increased the mRNA levels of LPS binding protein (*Lbp*), *Cd14*, and argininosuccinate synthase 1 (*Ass1*), which were all reversed by IFN-γ treatment (Figure 2E). Meanwhile, IFN-γ treatment also decreased the production of alcohol-induced chemokines, including *Cxcl1* and *Mcp1* (Figure 2E). IFN-γ treatment, however, did not improve plasma ALT and AST levels (ALT levels: PF 15.3 ± 4.2 U/L vs. AF 70.0 ± 8.9 U/L, *p* < 0.01, AF vs. AF+IFN-γ 62.9 ± 8.4 U/L, *p* = 0.125; AST levels: PF 19.7 ± 4.0 U/L vs. AF 57.1 ± 8.3 U/L, *p* < 0.01, AF vs. AF+IFN-γ 63.2 ± 9.1 U/L, *p* = 0.538). Alcohol feeding significantly increased hepatic triglyceride (PF 25.2 ± 4.1 nmol/mg vs. AF 40.1 ± 8.6 nmol/mg, *p* < 0.01) and free fatty acid (PF 5.3 ± 2.5 nmol/mg vs. 17.7 ± 6.9 nmol/mg, *p* < 0.05) levels, whereas IFN-γ treatment did not have an impact on lipid compositions in the liver (42.6 ± 6.8 nmol/mg TG, *p* = 0.647, and 19.6 ± 4.9 nmol/mg FFAs, *p* = 0.329). It suggests that short term IFN-γ treatment has beneficial effects in ameliorating alcohol-induced hepatic inflammation, but not lipid accumulation, through reducing PAMP translocation.

### Restitution of IFN-γ Improves Alcohol-Impaired Gut Barrier and AMP Production

**Figure 3:**
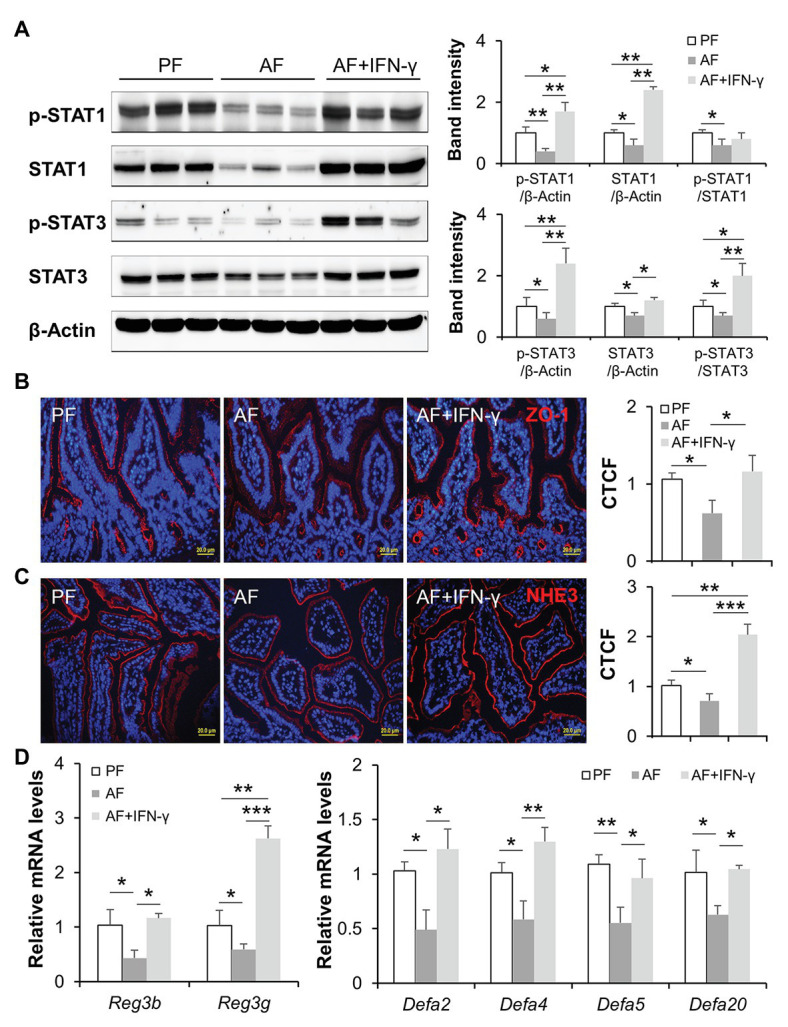
IFN-γ treatment activates intestinal STATs, improves gut barrier, and reversed alcohol-reduced antimicrobial peptide (AMP) levels. **(A)** WB and quantification of ileal STAT1 and STAT3. **(B)** IF staining of ileal ZO-1 (red) and nuclei (blue). Scale bar, 20 μm. Corrected total cell fluorescence (CTCF) of ZO-1 was quantified. **(C)** IF staining and quantification of ileal sodium-hydrogen exchanger 3 (NHE3; red). Nuclei were counterstained by DAPI (blue). Scale bar, 20 μm. **(D)** mRNA levels of ileal AMPs (*n* = 6 per group). ^*^p < 0.05, ^**^p < 0.01, and ^***^p < 0.001. PF, pair-fed; AF, alcohol-fed.

We further investigated gut barrier and AMP production to explore possible mechanisms of how IFN-γ treatment reduces alcohol-induced PAMP translocation. Alcohol-suppressed protein levels of p-STAT1, total STAT1, p-STAT3, and total STAT3 were reversed by IFN-γ treatment in mouse ileum to a level that were even higher than those in PF mice (Figure 3A). Disassembly of intestinal tight junction protein, ZO-1, was detected in AF mice compared to a continuous circumferential distribution of ZO-1 in PF mice, and IFN-γ treatment significantly improved ZO-1 distribution (Figure 3B). NHE3 is highly expressed at the apical part of differentiated IECs and used as a functional marker of IECs (Kozuka et al., 2017). We found that chronic alcohol feeding decreased the fluorescent intensity of ileal NHE3 compared to PF group and IFN-γ treatment dramatically reversed alcohol-reduced NHE3 (Figure 3C). In consistent with previous reports by our and others’ laboratory (Yan et al., 2011; Wang et al., 2016; Zhong et al., 2019), expression of small intestinal AMPs, including Reg3β, Reg3γ produced by IECs and Paneth cells, and α-defensins produced exclusively by Paneth cells, were all decreased by alcohol and reversed by IFN-γ treatment (Figure 3D). Thus, IFN-γ treatment restored intestinal STAT signaling, allowing repair of alcohol-damaged gut barrier and regulation of AMP production.

### IFN-γ Orchestrates Gut Microbiota Composition and Microbial Functional Pathways

**Figure 4:**
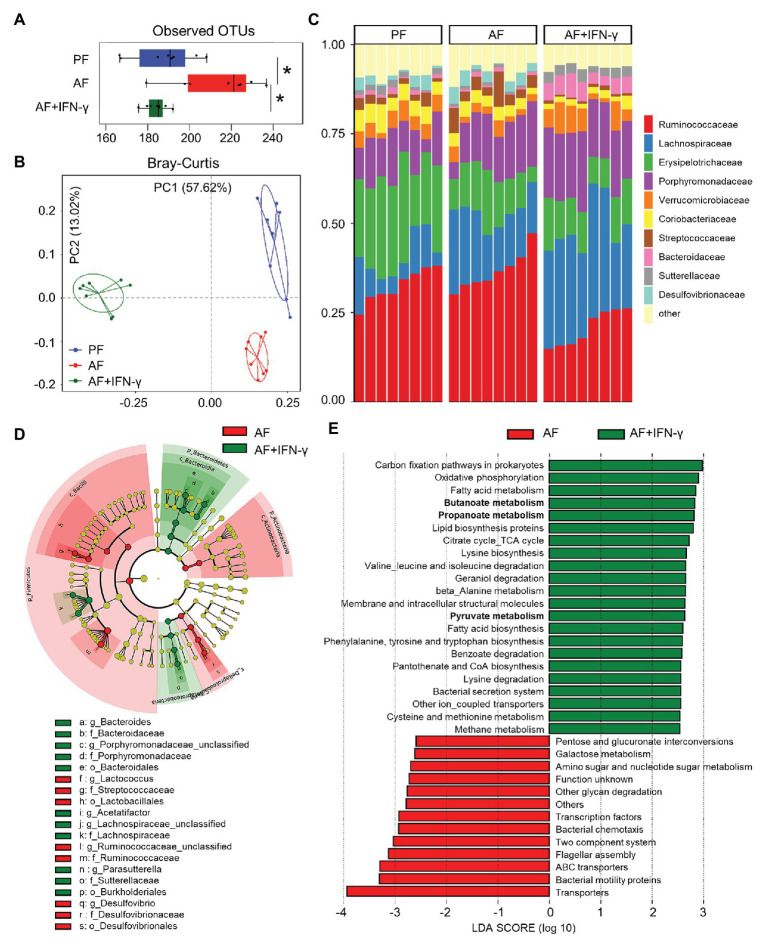
IFN-γ treatment ameliorates chronic alcohol exposure-induced cecal microbial dysbiosis in mice. **(A)** Alpha-diversity measurement of observed number of operational taxonomic units (OTUs). **(B)** PCoA plot showing dissimilarity in bacterial community structures based on Bray-Curtis distances. **(C)** Barplot showing the bacterial composition at family level. **(D)** Cladogram taxonomic abundances of bacteria. Legend of prominent taxons is shown at bottom. **(E)** Bacterial gene functions predicted based on 16S rRNA gene sequences using the PICRUSt algorithm and annotated from KEGG databases (*n* = 8 per group). ^*^p < 0.05. PF, pair-fed; AF, alcohol-fed.

Due to the intimate relationship between AMPs and gut microbiota homeostasis, we hypothesized that IFN-γ treatment would improve alcohol-induced gut microbial dysbiosis in association with upregulated AMP expression. Cecal microbiome was analyzed by metagenomic sequencing of the 16S rRNA gene. Compared with PF group, the observed OTUs index, an α-diversity value showing overall microbial richness, was significantly higher in AF group and normalized in AF+IFN-γ group (Figure 4A). Bray-Curtis distance matrices, which evaluate phylogenetic similarities between microbial communities, were used to calculate β-diversity; the three groups were clearly separated into different clusters (ANOSIM, *p* < 0.001, *r* = 0.926), indicating distinct gut microbial community structures among these groups (Figure 4B). Despite different microbial richness and diverse communities, IFN-γ treatment clearly affected the gut microbial configuration. At the family level, the relative abundances of *Verrucomicrobiaceae* and *Sutterellaceae* were significantly lower in AF group than in PF group (*p* = 0.019 and *p* = 0.008, respectively), which were restored by IFN-γ treatment (*p* = 0.036 and *p* < 0.001, respectively). Meanwhile, the relative abundance of *Streptococcaceae* was significantly higher in AF group than in PF group (*p* = 0.045) and was diminished by IFN-γ treatment (*p* = 0.002; Figure 4C). To further identify the distinguishing phylotypes in the gut microbiota between AF and AF+IFN-γ mice, we performed LEfSe analysis based on the taxonomy data. Cladogram in Figure 4D shows that the microbial structure of AF+IFN-γ mice was characterized by preponderant *Bacteroides*, *Bacteroidaceae*, *Porphyromonadaceae*, *Lachnospiraceae*, *Parasutterella*, *Sutterellaceae*, and *Burkholderiales*, and reduced *Lactococcus*, *Streptococcaceae*, *Ruminococcaceae*, *Desulfovibrio*, and *Desulfovibrionales* abundance [LDA score (log_10_) > 4.0; red, IFN-γ-decreased; green, IFN-γ-increased].

Apart from phylogenic insights, metagenomic analysis also provided an opportunity to assess the functional potential associated with the microbial community. We performed PICRUSt analysis of the microbiome and defined 35 dominant Kyoto Encyclopedia of Genes and Genomes (KEGG) pathways that were significantly different between the two AF groups (Figure 4E). Many microbial genes that could potentially trigger inflammatory responses, such as LPS biosynthesis (amino sugar and nucleotide sugar metabolism), bacterial motility (bacterial motility proteins), communication (two component system), chemotaxis, and flagellar assembly, were predicted to be inhibited by IFN-γ compared to that of AF group. On the other hand, the relative abundance of 22 KEGG pathways in AF+IFN-γ group was higher than in AF group. This includes bacterial genes involved in the metabolism of proteins, amino acids, carbohydrates, and fatty acids. Of great interest is the genes involved in short chain fatty acid (SCFA) metabolism, including butanoate metabolism, propanoate metabolism, and pyruvate metabolism (Figure 4E; bolded), suggesting that IFN-γ not only inhibits overgrowth of pathogenic bacteria but also drives gut microbiota toward a beneficial direction to improve ALD with the possible involvement of microbial-derived metabolites, such as SCFAs.

### IFN-γ Directly Regulates Intestinal AMPs Through STAT Signaling

**Figure 5:**
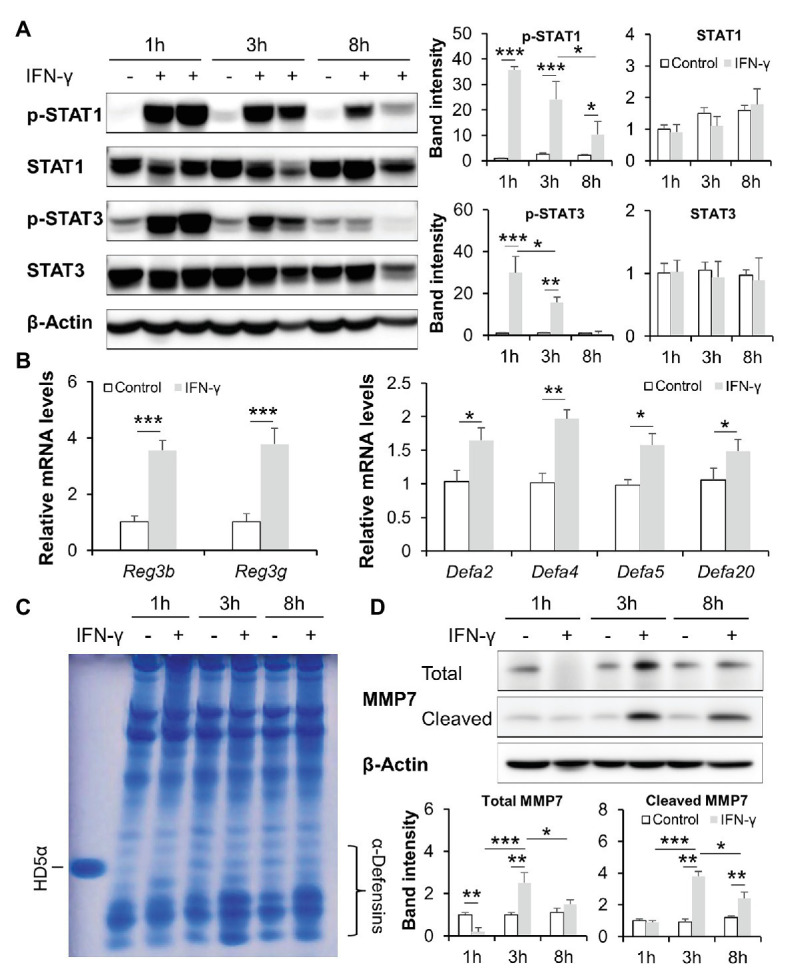
IFN-γ time dependently activates intestinal STATs and stimulates AMPs in mice. **(A)** WB and quantification of ileal STAT1 and STAT3. **(B)** mRNA levels of ileal AMPs. **(C)** Acid urea polyacrylamide gel electrophoresis (AU-PAGE) of freshly isolated ileal peptides. Synthetic human α-defensin 5 (HD5) was included as a control. **(D)** WB and quantification of total and cleaved matrix metallopeptidase 7 (MMP7) in the ileum of mice (*n* = 3 per group). ^*^p < 0.05, ^**^p < 0.01, and ^***^p < 0.001.

We next tested the effects of IFN-γ in regulating intestinal AMPs in an acute time-course IFN-γ treatment study. As shown in Figure 5A, one dose of IFN-γ treatment induced robust phosphorylation of both STAT1 and STAT3 in mouse ileum; the induction lasted for up to 8 h with 1 h being the most significant time point. At 8 h, the expression of Reg3β and Reg3γ was upregulated in IFN-γ-treated mice compared to the control. IFN-γ treatment also led to increased mRNA levels of α-defensins, including *Defa2*, *Defa4*, *Defa5*, and *Defa20* (Figure 5B). The presence and activation state of α-defensins in the lumen of ileum was directly assessed by AU-PAGE. Extracts from freshly isolated ileal peptides contained α-defensin species with mobilities consistent with HD5. Compared with the controls, the levels of active α-defensins were gradually increased by IFN-γ in a time-dependent manner (Figure 5C). MMP7, which is responsible for the activation of pro-α-defensins (Wilson et al., 1999), was also induced by IFN-γ, with the most significant induction at 3 h (Figure 5D).

**Figure 6:**
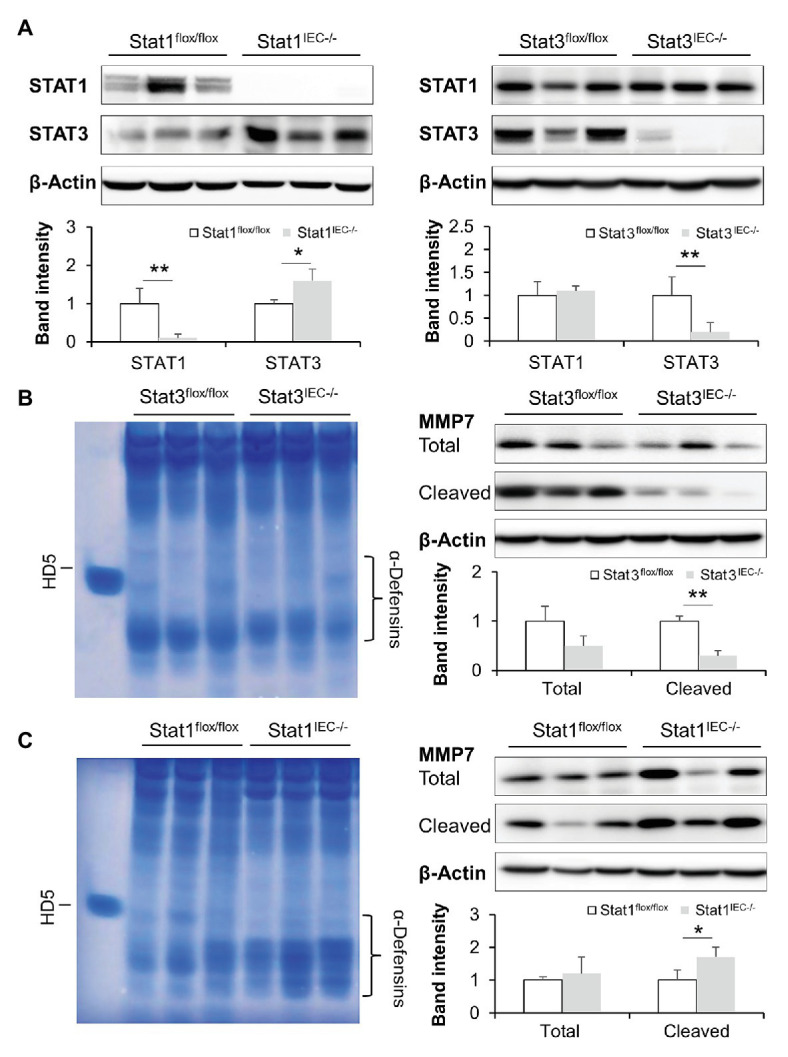
Intestinal epithelial STAT1 and STAT3 regulate MMP7 and activation of α-defensins. **(A)** WB and quantification of STAT1 and STAT3 in the ileum of Stat1^IEC−/−^ and Stat3^IEC−/−^ mice. **(B)** AU-PAGE of freshly isolated ileal peptides, and WB and quantification of MMP7 in control and Stat3^IEC−/−^ mice. **(C)** AU-PAGE of freshly isolated ileal peptides, and WB and quantification of MMP7 in control and Stat1^IEC−/−^ mice (*n* = 3 per group). ^*^p < 0.05 and ^**^p < 0.01.

Mice with IEC-specific deletion of STAT1 or STAT3 were generated to dissect the role of STATs in mediating IFN-γ-regulated intestinal AMPs. Immunoblotting confirmed successful deletion of corresponding STAT proteins in the knockout (KO) models (Figure 6A). With STAT1 deletion, the levels of intestinal STAT3 were slightly increased compared to floxed controls, whereas KO of STAT3 did not affect STAT1 levels. We first compared the levels of active α-defensins in these mice. Deletion of STAT3 in IECs decreased the levels of active α-defensins compared to the control. Meanwhile, cleaved MMP7, the active form that cleaves pro-α-defensins to active α-defensins, was dramatically declined in Stat3^IEC−/−^ mice (Figure 6B). IEC-specific deletion of STAT1, however, led to increased levels of intestinal active α-defensins and elevated levels of cleaved MMP7 (Figure 6C).

**Figure 7:**
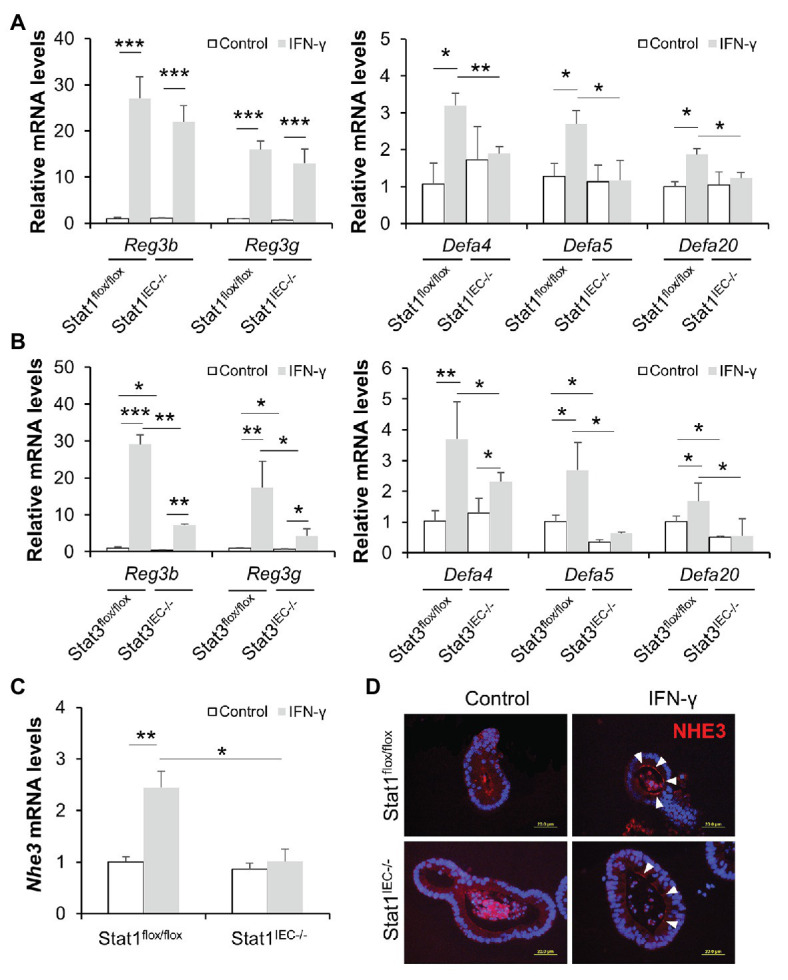
IFN-γ directly stimulates AMP expression in cultured organoids through STAT pathway. **(A)** mRNA levels of AMPs in organoids isolated from control and Stat1^IEC−/−^ mice after IFN-γ treatment. **(B)** mRNA levels of AMPs in organoids isolated from control and Stat3^IEC−/−^ mice after IFN-γ treatment. **(C)** mRNA levels of *Nhe3* (*n* = 3 per group). **(D)** IF staining of NHE3 (red) in organoids. Nuclei were counterstained by DAPI (blue). Scale bar, 20 μm. ^*^p < 0.05, ^**^p < 0.01, and ^***^p < 0.001.

To exclude the possible interference of immune cells and microbiota in IFN-γ-STAT signaling, we established small intestinal organoid cultures isolated from floxed control and IEC-specific STAT KO mice and treated them with IFN-γ. IFN-γ triggered more than 20-fold elevation in mRNA levels of Reg3β and Reg3γ in organoids isolated from floxed mice; it also significantly upregulated expression of *Defa4*, *Defa5*, and *Defa20* (Figures 7A,B). Lack of STAT1 prevented IFN-γ-induced α-defensin expression but not Reg3 expression (Figure 7A), whereas STAT3 deficiency impaired the upregulation of both Reg3 and α-defensins caused by IFN-γ (Figure 7B). Of note, deletion of STAT3 *per se* resulted in lower levels of *Reg3b*, *Reg3g*, *Defa5*, and *Defa20* even without IFN-γ stimulation, indicating an essential role of STAT3 in maintaining the expression of these AMPs. Moreover, we found that IFN-γ was capable of inducing NHE3 expression in cultured organoids isolated from floxed control mice, which was diminished by KO of IEC STAT1 (Figure 7C). IF staining showed increment in NHE3 positive staining after IFN-γ treatment in floxed group, whereas the induction was much weaker in Stat1^IEC−/−^ group (Figure 7D). Therefore, through *in vivo* and *in vitro* experiments, we identified an important role of IFN-γ in regulating intestinal AMPs *via* STAT signaling.

### IL-18 Treatment Restores Intestinal IFN-γ Levels and Ameliorates Alcohol-Induced Liver Damage

**Figure 8:**
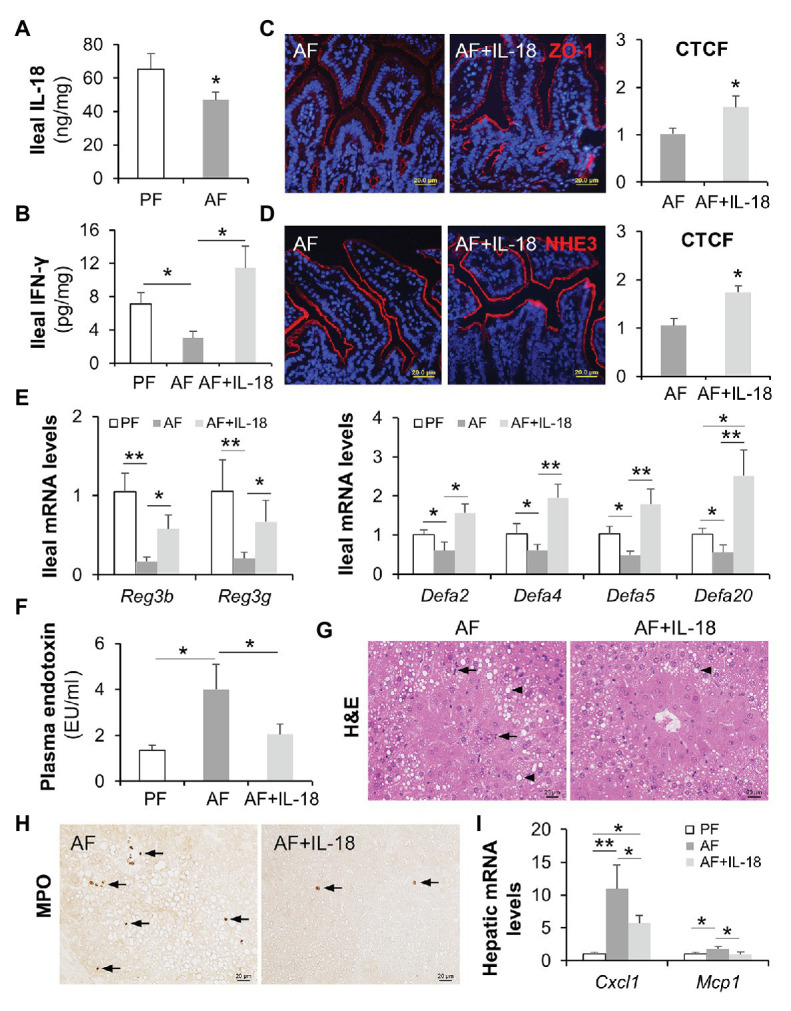
IL-18 treatment restitutes intestinal IFN-γ levels and ameliorates alcohol-induced gut-liver damage in mice. **(A)** Ileal IL-18 levels measured by ELISA. **(B)** Ileal IFN-γ levels. **(C)** IF staining and quantification of ileal ZO-1 (red). Nuclei were counterstained by DAPI (blue). Scale bar, 20 μm. **(D)** IF staining and quantification of ileal NHE3 (red). Nuclei were counterstained by DAPI (blue). Scale bar, 20 μm. **(E)** mRNA levels of ileal AMPs. **(F)** Plasma endotoxin levels. **(G)** H&E staining of mouse liver sections. Arrowheads indicate lipid accumulation and arrows indicate inflammatory cells. Scale bar, 20 μm. **(H)** Representative IHC staining of neutrophil marker, MPO, in the liver of mice. Arrows indicate positive staining. Scale bar, 20 μm. **(I)** mRNA levels of hepatic inflammatory chemokines, *Cxcl1* and *Mcp1* (*n* = 6 per group). ^*^p < 0.05 and ^**^p < 0.01. PF, pair-fed; AF, alcohol-fed; CTCF, corrected total cell fluorescence.

It was not fully understood how alcohol intoxication leads to suppressed intestinal IFN-γ production, so we explored the levels of IFN-γ inducing factor (interleukin-18/IL-18; Nakamura et al., 1989) after chronic alcohol feeding and the effects of IL-18 in restoring intestinal IFN-γ levels as well as in reversing the pathogenesis of ALD. Compared with PF mice, AF mice had lower levels of IL-18 in the ileum (Figure 8A). Recombinant mouse IL-18 was then given to AF mice for the last 2 weeks in an 8-week feeding experiment. As expected, IL-18 treatment effectively restored alcohol-declined ileal IFN-γ levels (Figure 8B), without affecting the levels of IL-22 (AF 21.66 ± 11.66 pg/mg vs. AF+IL-18 10.13 ± 4.54 pg/mg, *p* = 0.084, compared to PF 59.47 ± 9.46 pg/mg, *p* < 0.001) which is known to be decreased by alcohol and could protect against ALD (Ki et al., 2010; Hendrikx et al., 2019). Intestinal barrier function was significantly improved by IL-18 as indicated by refined distribution of tight junction protein ZO-1 (Figure 8C) and enhanced expression of NHE3 (Figure 8D). In association with restored IFN-γ levels, IL-18-treated AF mice had higher levels of ileal *Reg3b* and *Reg3g* than AF mice. IL-18 treatment also reinstated alcohol-reduced expression of α-defensins (Figure 8E). In accordance with improved gut barrier and antimicrobial ability, AF mice received IL-18 exhibited lower plasma endotoxin levels than AF mice (Figure 8F). Alcoholic liver damage was compared between the two AF groups. IL-18 treatment reduced alcohol-elevated plasma ALT and AST levels (ALT levels: AF 78.9 ± 16.6 U/L vs. AF+IL-18 41.8 ± 15.4 U/L, *p* < 0.05; AST levels: AF 62.9 ± 14.3 U/L vs. AF+IL-18 35.4 ± 11.1 U/L, *p* < 0.05). IL-18 treatment ameliorated alcohol-induced hepatic inflammation as evidenced by reduced inflammatory cell infiltration (Figure 8G, arrows), less positive staining of neutrophil marker MPO (Figure 8H, arrows), and diminished expression of inflammatory chemokines *Cxcl1* and *Mcp1* (Figure 8I). Similar to IFN-γ treatment, IL-18 treatment did not further alter hepatic TG or FFA levels (TG levels: AF 46.1 ± 11.6 nmol/mg vs. AF+IL-18 40.6 ± 8.8 nmol/mg, *p* = 0.531; FFA levels: AF 15.1 ± 4.6 nmol/mg vs. AF+IL-18 16.6 ± 5.8 nmol/mg, *p* = 0.255). These results suggest that IL-18 treatment is potent in restoring intestinal IFN-γ levels and treating ALD.

## Discussion

**Figure 9:**
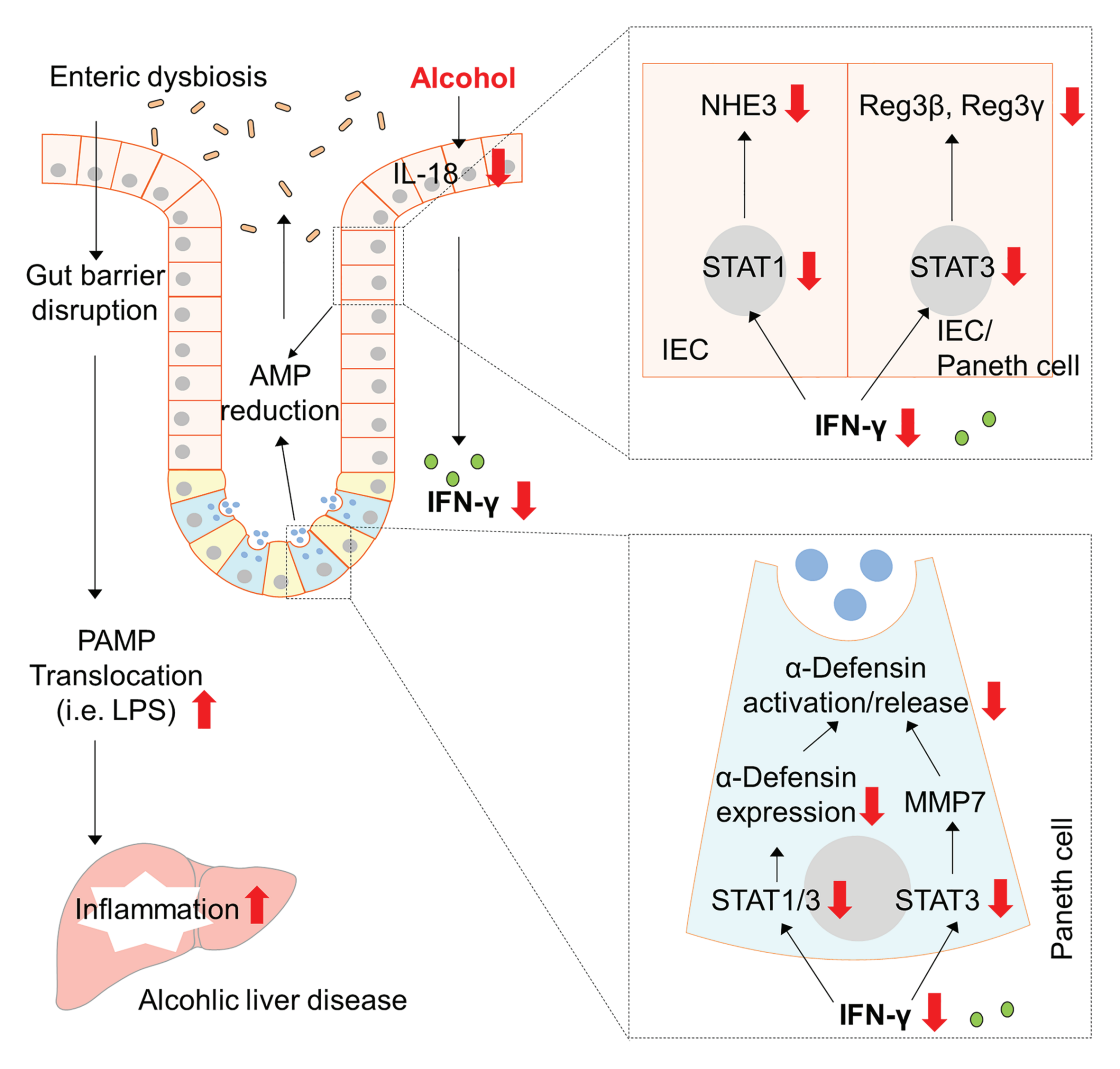
Schematic diagram about the major findings of the present study. Red arrows indicate changes caused by alcohol. AMP, antimicrobial peptide; IFN-γ, interferon gamma; IEC, intestinal epithelial cells; IL-18, interleukin-18; LPS, lipopolysaccharide; MMP7, matrix metallopeptidase 7; NHE3, sodium-hydrogen exchanger 3; PAMP, pathogen-associated molecular pattern; STAT, signal transducer and activator of transcription.

Sustained gut microbial dysbiosis causes disequilibrium in energy homeostasis, disrupted gut barrier, as well as stress and inflammatory responses that ultimately leads to metabolic diseases, including ALD (Mutlu et al., 2012; Tilg et al., 2016). Human and animal studies show that modulation of gut microbiota seems to be a promising strategy to reduce alcohol-induced liver injury (Philips et al., 2017; Grander et al., 2018). Nevertheless, the precise mechanisms by which alcohol causes gut dysbiosis and the subsequent liver damage are still poorly understood. Our laboratory and others’ have previously reported that alcohol intoxication hampers the antimicrobial ability of the host through reducing AMP production (Yan et al., 2011; Wang et al., 2016; He et al., 2019; Zhong et al., 2019). The present study revealed an essential role of intestinal IFN-γ-STAT signaling in regulating AMP levels, gut microbiota homeostasis, and alcohol-induced liver injury in mice (major findings summarized in Figure 9). We found that chronic alcohol feeding caused aberrant IFN-γ-STAT signaling in the intestine, which is responsible for maintaining AMP levels and gut microbiota symbiosis. We further demonstrate that IL-18 acts as an upstream regulator of IFN-γ and treatment of IL-18 restores intestinal IFN-γ levels and ameliorates liver injury induced by alcohol.

It is well-known that IFN-γ can enhance the innate immune response to epithelial cells and can boost the proinflammatory response of lymphocytes. It has been reported that IFN-γ strongly increased proliferation of intestinal epithelial T84 cells peaked at 24 h and substantially increased apoptosis from 24 to 72 h, which suggests that the effect of IFN-γ on epithelial proliferation is bidirectional and dose/time-dependent (Nava et al., 2010). There is mounting evidence, however, showing that IFN-γ also plays a protective role in a number of disease models (Ma et al., 2011). It has been reported that IFN-γ deficiency exacerbates inflammatory bowel disease in mice (Sheikh et al., 2010). Mechanically, IFN-γ can defend intestinal epithelium through upregulating cellular methylation pathways (Kominsky et al., 2011), inducing expression of indoleamine 2, 3-dioxygenase 1 (IDO1; Gurtner et al., 2003), and maintaining epithelial IL-10 signaling (Kominsky et al., 2014). This study aimed to define the role of IFN-γ in mediating the antimicrobial ability of the host. We found that IFN-γ regulates a broad spectrum of AMPs at both transcriptional and post-translational levels. Through utilizing IEC-specific STAT1 or STAT3 KO mice models, we then elucidated that the effects of IFN-γ in regulating intestinal AMPs are through differential STAT1 and/or STAT3 signaling. We proved that restitution of IFN-γ levels by IFN-γ or IL-18 treatment ameliorated alcohol-induced gut and liver inflammation. This concept is not without precedence. IFN-γ has previously been reported to protect against bacterial and viral infections (Ma et al., 2011; Thiemann et al., 2017), regulate cathelicidin expression (Shtrichman and Samuel, 2001; Fabri et al., 2011), and directly stimulate AMP and mucus release (Farin et al., 2014). However, it remains unclear what is the turning point that directs IFN-γ toward protective or pathogenic directions. We presume that the intensity of IFN-γ signal, the condition of intestinal epithelium, and microenvironment cues inference with each other and all impact on the outcome. The goal of the present study was to restore intestinal IFN-γ to normal levels, and all data discussed are within this scope. It is noteworthy to mention that alcohol *per se* can directly disrupt the barrier of the intestine, such as colon (Zhong et al., 2010; Mutlu et al., 2012), and induce PAMP translocation. The present study, however, did not compare hepatic and systemic ethanol and acetaldehyde levels. Moreover, we found that neither IFN-γ nor IL-18 treatment altered major hepatic lipid compositions, including triglycerides and free fatty acids. Although inflammation and lipid metabolism are intertwined modulators of homeostasis and immunity, it is still obscure which specific lipid species would be impacted by inflammatory mediators given broad lipid categories, including triglycerides, free fatty acids, sterols, and phospholipids. Besides, we only treated the mice with IFN-γ or IL-18 for a short period during the 8-week alcohol feeding, and that may not be long enough to reverse lipid accumulation in the liver. One of the most interesting findings in this study is that IFN-γ orchestrates gut microbiota composition and possibly microbial-derived metabolites to an extend far beyond our current knowledge. First, IFN-γ treatment reduces overall gut microbial richness that is expanded by alcohol intoxication, which is in line with stimulated AMP levels and the well-known antimicrobial function of AMPs. Second, it seems that IFN-γ specifically targets certain bacterial species rather than simple “correction” of a whole spectrum of microbiota. For example, IFN-γ treatment led to a proportional increase of *Verrucomicrobiaceae*. We previously reported that treatment of synthetic HD5 to AF mice resulted in a strikingly enriched *Verrucomicrobiaceae* population (Zhong et al., 2019). Similarly, another study also reported that HD5 increased *Akkermansia* sp. (the major genus of *Verrucomicrobiaceae*) without affecting microbial diversity in mice (Ehmann et al., 2019). Of note, *Akkermansia muciniphila* has been reported to be reduced upon alcohol intoxication, and treatment of *A. muciniphila* promotes gut barrier function and protects against ALD in mice (Grander et al., 2018). In addition, we also found that IFN-γ reversed alcohol-enriched *Streptococcaceae* and alcohol-diminished *Sutterellaceae*, which were not observed in HD5 treated mice, suggesting a HD5-independent regulatory mechanism of gut microbiota by IFN-γ. Third, functional pathway analysis of microbial genes revealed that IFN-γ also impacts on microbial metabolism, especially the production of SCFAs. SCFAs, especially butyric acid, are of great importance for their energy supply (Schonfeld and Wojtczak, 2016). They are also involved in intestinal epithelial AMP productions (Zhao et al., 2018; Pearce et al., 2020). This may partially explain the aforementioned beneficial effects of IFN-γ in regulating intestinal homeostasis and needs to be taken into consideration when interpreting data of the *in vivo* treatment experiments in the present study. We, therefore, used *in vitro* organoids and validated that IFN-γ can directly stimulate AMP expression in the absence of microbial-derived metabolites.

Although it has been established that IFN-γ-mediated signal transduction is mainly through Janus kinase (JAK)-STAT1 pathway, emerging evidence show that STAT1-independent pathways also play important roles in a wide range of biological responses of IFN-γ (Ramana et al., 2002; van Boxel-Dezaire and Stark, 2007). According to previous reports, STAT3 competes with STAT1 for activation at the same motif of IFN-γ receptor subunit 1 (IFNGR1; Qing and Stark, 2004). Moreover, IFN-γ-induced STAT3 activation was stronger and more prolonged in STAT1 deficient mouse embryo fibroblasts than in WT cells (Qing and Stark, 2004). The present study shows that both one and repeated administration of IFN-γ lead to substantial activation of intestinal STAT1 and STAT3. We further demonstrate that genetic deletion of IEC STAT1 causes STAT3 induction. This is not surprising as it has been reported that STAT1 may suppress STAT3 and compete it in many biological aspects, such as inflammation and tumorigenesis (Hu and Ivashkiv, 2009). We then dissected the roles of IEC STAT1 and STAT3 in mediating IFN-γ-regulated AMP levels and gut barrier. STAT3 is involved in the expression of Reg3 and α-defensins as well as the activation of α-defensins *via* activating MMP7, whereas STAT1 mediates the expression of α-defensins and NHE3. These synergistic interactions demonstrate that cross-regulation of STAT signaling processes can lead to a broad-spectrum of host response. These observations on the regulation of AMPs and gut microbiota by IFN-γ-STAT signaling indicate an essential role of IFN-γ-STAT signaling in intestinal homeostasis under normal and disease conditions.

IFN-γ is produced by a variety of immune cells, including CD4^+^ T helper 1 (Th1) cells, natural killer (NK) cells, NK T cells, neutrophils, and macrophages (Beaurepaire et al., 2009). Alcohol abuse has been shown to be accompanied by suppressed immune response (Sibley et al., 2001). Alcohol abuse suppresses NK cell activity and decreases NK cell numbers (Meadows et al., 1989; Zhang et al., 2011). Moreover, in a mouse model of a combined insult of alcohol and burn injury, gut-associated lymphoid T cells are suppressed, intestinal IFN-γ is reduced, and bacterial translocation is elevated in association with exaggerated disease progression (Choudhry et al., 2002; Li et al., 2014). Here, we explored an epithelial-derived regulatory mechanism by IL-18 in regulating and maintaining physiological levels of IFN-γ. IL-18 was originally discovered as a cytokine that induces IFN-γ production by Th1 cells (Nakamura et al., 1989). Under homeostatic conditions, intestinal IL-18 is involved in epithelial cell repair, proliferation, and maturation (Wlodarska et al., 2014; Yasuda et al., 2019). The present study demonstrates that chronic alcohol feeding reduces IL-18 and administration of IL-18 restores intestinal IFN-γ levels (but not IL-22), reverses alcohol-reduced AMP expression, and alleviates alcoholic liver damage. In rats challenged with alcohol gavage and burn injury, however, a significant increase in IL-18 levels was observed, whereas alcohol gavage only did not change IL-18 levels (Li et al., 2006). This discrepancy may have resulted from different rodent species and disease models, administration routines, and the given dosages.

In summary, the present study demonstrates that constitutive expression of IFN-γ is instrumental in maintaining intestinal STAT signaling, innate immune responses of IECs, and gut microbial symbiosis to combat alcohol toxicity at the gut-liver axis, leading to a better control of the pathogenesis upon alcohol intoxication. Additionally, these findings provide new understanding of how the lack of adequate IFN-γ-STAT signaling may fail to elicit antimicrobial responses of IECs and thus provide an opportunity for progression of ALD. Therefore, IFN-γ-based interventions, such as IL-18 treatment, could be considered as a way of boosting intestinal epithelial innate immunity to halt systemic PAMP translocation and may also be one approach to preventing the development of ALD.

## Data Availability Statement

Raw data were submitted to the National Center for Biotechnology Information (NCBI) Short Read Archive Database and are available with BioProject accession number PRJNA688787.

## Ethics Statement

The animal study was reviewed and approved by North Carolina Research Campus Institutional Animal Care and Use Committee.

## Author Contributions

WZ conceived and designed research. RY, ZZ, and WZ performed experiments and analyzed data. XW and JZ analyzed gut microbiota and prepared figures. RY prepared figures and drafted manuscript. All authors contributed to the article and approved the submitted version.

### Conflict of Interest

The authors declare that the research was conducted in the absence of any commercial or financial relationships that could be construed as a potential conflict of interest.

## Footnotes

^1^http://huttenhower.sph.harvard.edu/galaxy/
